# Identification and Characterization of the Heat-Induced Plastidial Stress Granules Reveal New Insight Into *Arabidopsis* Stress Response

**DOI:** 10.3389/fpls.2020.595792

**Published:** 2020-10-30

**Authors:** Monika Chodasiewicz, Ewelina Maria Sokolowska, Anna C. Nelson-Dittrich, Aleksandra Masiuk, Juan Camilo Moreno Beltran, Andrew D. L. Nelson, Aleksandra Skirycz

**Affiliations:** ^1^Max Planck Institute of Molecular Plant Physiology, Golm, Germany; ^2^Center for Desert Agriculture, Biological and Environmental Science and Engineering Division (BESE), King Abdullah University of Science and Technology (KAUST), Thuwal, Saudi Arabia; ^3^Boyce Thompson Institute, Cornell University, Ithaca, NY, United States

**Keywords:** stress granules, plastid, protome, chlorophyll biosynthesis, metabolites

## Abstract

Plants exhibit different physiological and molecular responses to adverse changes in their environment. One such molecular response is the sequestration of proteins, RNAs, and metabolites into cytoplasmic bodies called stress granules (cSGs). Here we report that, in addition to cSGs, heat stress also induces the formation of SG-like foci (cGs) in the chloroplasts of the model plant *Arabidopsis thaliana*. Similarly to the cSGs, (i) cpSG assemble rapidly in response to stress and disappear when the stress ceases, (ii) cpSG formation is inhibited by treatment with a translation inhibitor (lincomycin), and (iii) cpSG are composed of a stable core and a fluid outer shell. A previously published protocol for cSG extraction was successfully adapted to isolate cpSG, followed by protein, metabolite, and RNA analysis. Analogously to the cSGs, cpSG sequester proteins essential for SG formation, dynamics, and function, also including RNA-binding proteins with prion-like domain, ATPases and chaperones, and the amino acids proline and glutamic acid. However, the most intriguing observation relates to the cpSG localization of proteins, such as a complete magnesium chelatase complex, which is involved in photosynthetic acclimation to stress. These data suggest that cpSG have a role in plant stress tolerance.

## Introduction

Cell compartmentalization is a way to separate and organize biochemical reactions in order to increase the efficiency of cellular processes. In addition to the membrane-based compartmentalization characteristic for eukaryotic cells, recent evidence has shown that cells can sequester and organize biomolecules, proteins, nucleic acids, and metabolites in non-membrane compartments using the process of liquid–liquid phase separation (LLPS). LLPS is driven by the collective protein–protein and protein–nucleic acid interactions, leading to the emergence of reversible biological condensates (Courchaine et al., 2016; Alberti, 2017; Gomes and Shorter, 2019). Stress granules (SGs) are an example of an evolutionarily conserved non-membrane compartment formed in response to stress and are composed of cytosolic proteins, mRNAs, and small molecules (Protter and Parker, 2016; Chantarachot and Bailey-Serres, 2018; Youn et al., 2019).

The key to SG formation is self-assembly of intrinsically disordered proteins (IDPs). Prion-like domains provide structural flexibility and polymer-like behavior to IDPs, which enables the formation of biological scaffolds (Gilks et al., 2004; Weber et al., 2008). Key IDPs involved in SG assembly also contain RNA binding motifs that contribute to RNA sequestration within SGs (Gilks et al., 2004; Weber et al., 2008; Sorenson and Bailey-Serres, 2014). *Via* a network of both direct and indirect interactions, SG protein–RNA scaffolds attract numerous additional proteins (Markmiller et al., 2018; Youn et al., 2018) and, as recently shown, also small molecules (Kosmacz et al., 2019). SG-associated proteins can be classified into two classes: conserved and specific (Jain et al., 2016; Youn et al., 2018; Kosmacz et al., 2019). Conserved proteins are found across multiple SG isolations and represent functional classes required for SG assembly and dynamics, including the already mentioned IDPs, protein and RNA chaperones, ATPases, elongation initiation factors, and small ribosomal subunits. In contrast, sequestration of the specific proteins is dependent on organism, cell type, and developmental stage. Specific SG proteins include metabolic enzymes, e.g., *Arabidopsis* rhamnose synthase (Kosmacz et al., 2019) and regulators, such as kinases (Kosmacz et al., 2019), transcription factors (Bhasin and Hulskamp, 2017), and receptors (Lokdarshi et al., 2016).

The primary function postulated for SGs is the protection of mRNA and proteins from unfolding and degradation (Protter and Parker, 2016; Chantarachot and Bailey-Serres, 2018). Sequestration of signaling and regulatory proteins also rapidly inhibit their activity, with consequences for the cellular processes that depend on them. To illustrate this point, in yeast, TORC1, a key regulator of the cell cycle, is sequestered into SGs during heat stress, where it is maintained in an inactive state. During post-stress recovery, SGs disassemble and TORC1 is released, contributing to efficient growth restoration (Takahara and Maeda, 2012).

The most important characteristics of SGs are as follows (Protter and Parker, 2016; Chantarachot and Bailey-Serres, 2018; Youn et al., 2019): (i) SGs form under stress conditions associated with stalled translation, (ii) SGs sequester both proteins and mRNAs, (iii) SGs are characterized by the presence of conserved proteins involved in SG formation and dynamics, such as IDPs, (iv) SGs rapidly assemble under stress conditions and disassemble when the stress ceases, (v) because of their liquid-like properties, SGs can fuse with each other and also with other biological condensates such as processing bodies and in the process exchange components (Gutierrez-Beltran et al., 2015), and (vi) SGs are composed of a stable core and more dynamic, fluid shell.

Stress granules are historically associated with the cytosol; however, SG-like structures (chSGs) were also reported in the chloroplast of *Chlamydomonas reinhardtii*, a single-cell green alga (Uniacke and Zerges, 2008). Composed of both proteins and mRNAs, chSGs formed in response to oxidative stress and disassembled during stress recovery. Interestingly, the key protein for chSG formation and mRNA sequestration proved to be a large subunit of Rubisco. Moreover, the authors showed that mRNAs are not permanently trapped within chSGs but rather cycle between chSGs and polysomes, a mechanism contributing to the regulation of plastidial translation under stress conditions. Herein and, to our knowledge, for the first time, we report that, in response to heat, SG-like foci also form in the chloroplasts of higher plants. Plastidial SGs (cpSG) sequester proteins, mRNAs, and metabolites, including key photosynthetic regulators, indicative of the importance of cpSG for *Arabidopsis* stress response.

## Materials and Methods

### Preparation of 35S:SCO1-GFP *Arabidopsis* Lines

*Arabidopsis thaliana* Col-0 plants were transformed with *35S:SCO1-GFP* (SCO1-corresponds to snowy cotyledon 1, nuclear-encoded protein localized in chloroplast, known to regulate translation in chloroplasts) provided by Dr. Chanhong Kim using a previously described (Zhang et al., 2006) floral dip method. T0 seeds were screened for BASTA resistance. The green fluorescent protein (GFP) signal in T1 generation was confirmed using confocal microscopy and is in agreement with previously reported SCO1 cellular localization (Albrecht et al., 2006). All the presented experiments were performed on the pool of seeds obtained from multiple transformation events.

### Plant Growth Conditions and Heat Stress Treatment

*Arabidopsis thaliana 35S:SCO1–GFP* transgenic seedlings (1.5 mg seeds, c. 100 plants) were grown in sterile liquid cultures (100-ml Erlenmeyer glass flasks) in 35 ml half-strength Murashige and Skoog (1/2MS) liquid medium (Murashige and Skoog, 1962) with 1% sucrose. The seedlings were kept shaking for 7 days on orbital shakers with the speed of 130 rpm, in constant light (c. 80 μmol m^–2^ s^–1^) and temperature (22°C) conditions. After 1 week, the medium in all flasks was changed. After 10 days, heat stress treatment was performed by submitting the flasks to 42°C for 30 min in darkness, which aimed to induce cpSG formation. The control seedlings were kept at room temperature (RT) and in the light. cpSG formation was evaluated by confocal microscopy.

### Plastidial Stress Granule Isolation Protocol

The plastidial stress granule extraction protocol was modified from Kosmacz et al. (2019). Extraction was performed in triplicate, starting from the same pool of plant material. Then, 8 g of pulverized material from control or stress (dark/heat) plant material was homogenized using a pre-cooled mortar and pestle with 8 ml of lysis buffer [50 mM Tris–HCl pH 7.4, 100 mM potassium acetate, 2 mM magnesium acetate, 0.5% NP-40, 0.5 mM DTT, 1 mM NaF, 1 mM Na_3_VO_4_, protease inhibitor cocktail (Sigma P9599) and 1 U μl^–1^ RNasin (Promega N2615)]. The resulting slurry was centrifuged at 4,000 *g* for 10 min at 4°C, the supernatant was removed, and the pellet was resuspended in 6 ml of lysis buffer. The obtained suspension was divided into three to four samples. These were centrifuged at 14,000 *g* for 10 min at 4°C. The pellets were resuspended in 1 ml lysis buffer, vortexed, and centrifuged at 14,000 *g* at 4°C for 10 min. As in the previous step, the supernatant was discarded, and the pellets were resuspended in 1 ml of lysis buffer. After a final centrifugation at 850 *g* for 10 min at 4°C, the supernatant was checked for the presence of cpSG by confocal microscopy and used in the further purification steps. A 1-ml volume (120 μl per sample) of Dynabeads protein A (Thermo Fisher 10002D, Hennigsdorf, Germany) was equilibrated for 30 min at RT with 1 ml of diethyl pyrocarbonate (DEPC)-treated phosphate-buffered saline (PBS) buffer (on a rotating wheel). The beads were separated with a magnet and washed once with DEPC-treated PBS buffer for 5 min at 4°C and then three times with the lysis buffer for 5 min at 4°C. A 370-μl volume of the cpSG fraction supplemented with RNasin (1:100 final dilution) was incubated (on a rotating wheel) with 60 μl of equilibrated Dynabeads for 15 min at room temperature. The beads were then separated, and the supernatant was incubated (on a rotating wheel) in a new tube with 12.5 μl anti-GFP rabbit IgG antibody (Life Technologies A11122, Hennigsdorf, Germany) for 60 min at RT. The excess anti-GFP antibody was separated by centrifugation at 14,000 *g* for 15 min at 4°C. The supernatant was removed, and the pellet was resuspended in 500 μl of lysis buffer supplemented with an additional 5 μl of RNasin. This resuspension was mixed (on a rotating wheel) with 60 μl of equilibrated Dynabeads for 15 min at RT, followed by several washing steps: three times for 5 min with lysis buffer at 4°C. In the final step, the supernatant was removed, and the SGs remained attached to the beads. Attached to the beads, protein and metabolites were extracted using a methyl tert-butyl ether/methanol/water (MTBE) solvent protocol described previously (Giavalisco et al., 2011).

### GAPCP1-GFP Foci Extraction Protocol

GAPCP1-GFP (glyceraldehyde-3-phosphate dehydrogenase, enzyme of the glycolytic pathway) foci extraction was performed following the protocol for stress granules extraction described above. The only difference was the amount of starting material in which 3 g (instead of 8 g) of ground *Arabidopsis* (without stress) material was used. Proteins and metabolites were extracted according to Giavalisco et al. (2011).

### Metabolite and Protein Extraction

Proteins, lipids, and polar compounds were extracted from the beads using the MTBE method (Giavalisco et al., 2011) known to separate molecules into a pellet (proteins), an organic phase (lipid compounds), and an aqueous phase (polar compounds). Proteins and metabolites were dried for 4 h up to overnight, respectively, in a centrifugal evaporator and stored at −80°C until liquid chromatography–mass spectrometry analysis.

### Protein Preparation

Protein pellets with beads were resuspended in 50 μl of denaturation buffer (6 M urea and 2 M thiourea in 40 mM ammonium bicarbonate). Cysteine residues were reduced by the addition of 2.5 μl of 100 μM DTT for 30 min at RT, followed by alkylation with 2.5 μl of 300 mM iodoacetamide for 20 min in the dark. The proteins were enzymatically digested using LysC/Trypsin Mix (Promega) according to the manufacturer’s instructions. Next, peptides were desalted on C18 SepPack columns and dried using a centrifugal evaporator to about 2 to 3 μl. Dried peptides were stored at −80°C until measurement.

### Proteomics Measurements and Data Analysis of SCO1-GFP

Dried peptides were solubilized in a loading buffer (3% ACN, 0.1% FA) and measured on a Q Exactive HF coupled to an Easy nLC1000 UHPLC reverse-phase nano-liquid chromatography (Thermo Fisher Scientific, Hennigsdorf, Germany). The gradient ramped from 4 to 24% ACN over 20 min and then to 45% ACN over the next 15 min, followed by a 5-min washout with 80% ACN. The MS was run using a data-dependent MS/MS method with the following settings: full scans were acquired at a resolution of 120,000, AGC target of 3e6, maximum injection time of 50 ms, and an m/z ranging from 200 to 2,000. dd-MS2 scan was recorded at the 15,000 resolution with an AGC target of 1e5, maximum injection time of 150 ms, isolation window of 1.2 m/z, normalized collision energy 30, and dynamic exclusion of 20 s. Raw chromatograms were processed using the MaxQuant (Version 1.6.0.16, MPI of Biochemistry, Germany) software using the *Arabidopsis* TAIR database (Version 10, The Arabidopsis Information Resource, www.Arabidopsis.org).

### Proteomics Measurements and Data Analysis of GAPCP-GFP

Dried peptides were solubilized in a loading buffer (3% ACN, 0.1% FA) and measured on a Q Exactive HF (Thermo Fisher Scientific, Hennigsdorf, Germany) coupled to a reverse-phase nano-liquid chromatography ACQUITY UPLC M-Class system (Waters). The gradient ramped from 5 to 24% ACN over 7 min and then to 36% ACN over the next 6 min, followed by a 2-min washout with 75% ACN. The MS was run using a data-dependent MS/MS method with the following settings: full scans were acquired at a resolution of 60,000, AGC target of 3e6, maximum injection time of 50 ms, and an m/z ranging from 300 to 1,600. dd-MS2 scan was recorded at 30,000 resolution with an AGC target of 1e5, maximum injection time of 100 ms, isolation window of 1.4 m/z, normalized collision energy 27, and dynamic exclusion of 30 s. Raw chromatograms were processed using the MaxQuant (Version 1.6.0.16, MPI of Biochemistry, Germany) software using the *Arabidopsis* TAIR database (Version 10, The Arabidopsis Information Resource, www.Arabidopsis.org).

### Data Availability

The mass spectrometry proteomics data for SCO1-GFP and GAPCP-GFP were deposited to the ProteomeXchange Consortium *via* the PRIDE (Vizcaino et al., 2016) partner repository with the table identifier PXD018348.

### Metabolomic Analysis and Metabolite Annotation

Small molecules were separated by ultraperformance liquid chromatography and analyzed on an Exactive Orbitrap MS (Thermo Fisher Scientific) in positive and negative ionization modes, as described previously (Giavalisco et al., 2011). Data processing, including peak detection and integration and removal of isotopic peaks and chemical noise, was performed using Refiner MS 7.5 (GeneData). An in-house database of chemical compounds was used to annotate the obtained metabolic features (m/z at a given retention time), allowing 10 ppm and 0.1 min deviations from the reference compound mass and retention time, respectively.

### Cloning of Plastidial Proteins and Transient Infiltration

Selected genes FNR, PORC, CYP-20, and CHLI2 were cloned into the vector pGWB554 (Nakagawa et al., 2007) to overexpress RFP-tagged fusion protein, and *Agrobacterium tumefaciens* was transformed. Next, *A. tumefaciens* strain GV3101 harboring the construct of plastidial genes was used to transiently transform (Lee and Yang, 2006) leaves of 3-week-old rossettas expressing plastidial stress granule marker SCO1-GFP. All transient expression assays were performed on the independently grown plants. For each transformation event, three leaves from five independent plants were transformed. Then, 30 min of 42°C dark treatment was used to induce stress granule formation, which was then observed under a confocal microscope.

### Confocal Microscopy

To validate the cellular localization of SCO1-GFP, images were acquired using a Leica DM6000B/SP5 confocal laser scanning microscope (Leica Microsystems, Wetzlar, Germany) on leaves from seedlings growing on the liquid medium as mentioned above. The SCO1-GFP signal was visualized using excitation of 488 nm laser and emission between 500 and 520 nm. For the transient infiltration experiment, co-localization images of SCO1-GFP and plastidial proteins were taken using sequential scan mode between lines, allowing separation for the excitation of two fluorophores at once. RFP signal was excited with 561-nm laser, and emission was detected between 595 and 620 nm.

### 1,6-Hexanediol Treatment

cpSG were extracted using centrifugation steps from the SG extraction protocol. Prepared cpSG-enriched lysate was treated with 10% of 1,6-hexanediol and immediately observed under a confocal microscope. A total of 15 t-stacks were collected using 20 s of intervals for 5 min of total time as suggested in Peskett et al. (2018). Measurement of fluorescent intensity per selected cpSG area was performed using Fiji (Schindelin et al., 2012) and region of interest option.

### RNA Extraction From Purified cpSG

cpSG were purified using the SG isolation protocol. RNA was extracted from two technical replicates of cpSG isolated from control (RT) and heat-treated (42°C) samples as follows: to each tube of Dynabeads-purified cpSG, 100 μl of nuclease-free water was added, followed by an equal volume of acid phenol/chloroform/isoamyl alcohol (124:25:1, Invitrogen). The sample was vortexed for 20 s and centrifuged at 21,000 *g* for 5 min at room temperature. The top layer of this sample was added to a new tube, followed by 100 μl of chloroform. The sample was vortexed for 20 s and centrifuged at 21,000 *g* for 2 min at room temperature. The top layer (∼80 μl) of this sample was added to a new tube. RNA was precipitated by adding 1 μl of 20 μg/μl glycogen (Invitrogen), 10 μl of 3 M sodium acetate, pH 5.5, and 250 μl of 100% ethanol and incubating at −80°C for 4 h. The sample was centrifuged at 21,000 *g* for 30 min at 4°C, followed by two washes with 70% ethanol and centrifugation at 21,000 g for 10 min at 4°C. Pellet was resuspended in 7.5 μl of nuclease-free water and stored on ice until library preparation.

### Library Preparation and RNA Sequencing of RNA From Purified cpSG

RNA sequencing libraries were prepared using the YourSeq Duet (FT + ’-DGE) RNAseq library kit (Amaryllis Nucleics). Input material was RNA pellets (7.5 μl) from cpSG, which were not quantified beforehand due to the low amount of RNA expected to be present. The full-transcript library preparation protocol was used as described by the manufacturer without modification, except that 19 cycles of PCR were used to amplify the libraries instead of the 14 cycles described in the protocol. Index #1 (ATGATTGA) and index #3 (GCTATTCT) were added to control cpSG RNA samples, and index #2 (GACTGCCT) and index #4 (AGCGTTAC) were added to heat-treated cpSG RNA samples. Prior to sequencing, the prepared libraries were quantified with a Qubit fluorometer (Thermo Fisher) and the Qubit dsDNA HS assay kit to ensure that a sufficient quantity of library (>10 μl of 2 ng/μl per sample) was present. Libraries were pooled and sequenced using the MiSeq Nano kit (Illumina), with 300 bp of paired-end reads (2 × 150 bp) generated. Adapter trimmed reads were mapped to the *Arabidopsis* genome (Araport11; Cheng et al., 2017) using the RMTA workflow (v2.6.3; Peri et al., 2019) with HiSat2 and 0 FPKM filter options.

## Results

### Heat Stress Induces the Formation of Plastidial SGs

The protein composition of SG cores was first described in mammalian and yeast cells, using an SG isolation protocol combining differential centrifugation and affinity purification against SG marker proteins (Wheeler et al., 2017). This protocol was recently adapted to *Arabidopsis*, yielding a list of 118 cytosolic proteins sequestered within SGs during heat stress (Kosmacz et al., 2019). Intriguingly and in addition to the cytosolic proteins, the SG isolates contained 28 proteins with a reported plastidial localization. Since the liquid phase condensates such as SGs were shown to fuse and, in the process, exchange components (Gutierrez-Beltran et al., 2015), we decided to explore the possibility of SG-like foci forming in plastids. During lysate preparation, such hypothetical plastidial SGs (cpSG) would fuse with cytosolic SGs (cSGs). Supporting such a scenario, the list of 28 plastidial proteins contained RNA-binding proteins, ATPase, chaperones, and translation elongation factors functionally related to the known SG components (Supplementary Figure S1 and Supplementary Table S1).

**FIGURE 1:**
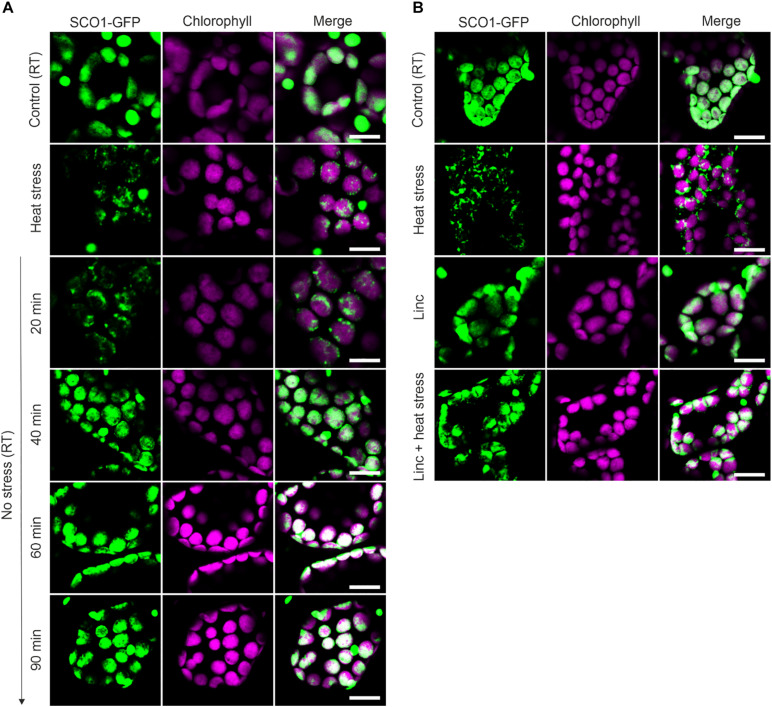
Dynamic of ccpSG assembly and disassembly visualized by the localization of SCO1-GFP signal in *Arabidopsis* leaves. **(A)** The localization of SCO1-GFP was followed under control (room temperature, RT) conditions, after 30 min of exposure to 42°C (heat stress) and during heat stress recovery (20, 40, 60, and 90 min). For each condition, three biological replicates were used (*n* = 3). **(B)** The localization of SCO1-GFP in plants treated with 50 μM lincomycin (Linc) for 1 h and subsequently 30 min of heat stress (Linc + heat stress). For each control and Linc treatment, three biological replicates were used (*n* = 3). **(A,B)** The green signal corresponds to GFP; magenta corresponds to auto-fluorescence of chlorophyll. Scale bars = 10 μm.

To examine the possibility of SGs forming in chloroplasts of higher plants, we expressed one of the 28 plastidial proteins identified in the cSGs, snowy cotyledon 1 (SCO1), as a fusion with GFP under the control of the constitutive cauliflower mosaic virus (CaMV) 35S promoter. This is *on par* with previous studies, where transgenic lines expressing cSG marker proteins, under the control of the constitutive promoter, were used to characterize the composition of the cSGs (Sorenson and Bailey-Serres, 2014; Kosmacz et al., 2019). SCO1 is a translation elongation factor (Albrecht et al., 2006), and interestingly, it shares homology with the translation elongation factor ELF5A-2 found to be present in the cSGs (Kosmacz et al., 2019). *In vitro*-grown 5-day-old 35S:aSCO1-GFP *Arabidopsis* seedlings were subjected to control and heat stress conditions (42°C, darkness, 30 min). While under control conditions, SCO1-GFP signal was detected uniformly in the plastidial stroma, in agreement with the previously published SCO1 localization (Albrecht et al., 2006); in response to heat, SCO1-GFP fluorescence turned into a dotted, star-like pattern reminiscent of the cSGs (Figure 1A). Importantly, in contrast to SCO1-GFP, the GFP protein expressed under the control of the 35S promoter showed no sign of aggregation under our experimental conditions (Kosmacz et al., 2019).

**FIGURE 2:**
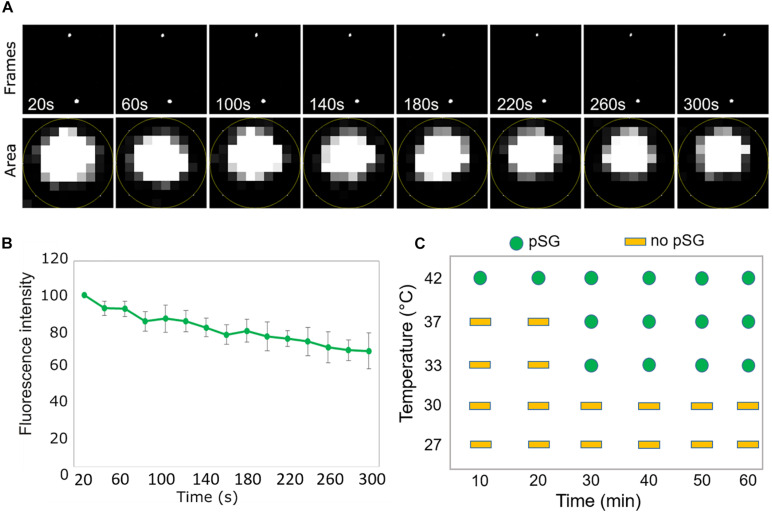
SCO1-GFP foci display SG-like properties. **(A)** Time scale analysis of a representative stack micrographs from 5 min of screen. The lower panel represents the region of interest defined area for mean gray value measurement (fluorescent intensity) for a single SCO1-GFP focus in the time scale frame. **(B)** Quantification of fluorescence intensity (from the lower panel of **(A)** of SCO1-GFP foci after addition of 10% 1,6-hexanediol. Data represent the mean of percentage (%) of fluorescent intensity per area of cpSG from five independent measurements of five independent extractions (*n* = 5, error bars = SD). Measurements are included in Supplementary Table S2. **(C)** Graphical representation of temperature- and time-dependent ccpSG formation in *Arabidopsis* seedlings expressing SCO1-GFP. Micrographs are included in Supplementary Figure S2.

We next decided to investigate whether, in addition to the heat-induced assembly, SCO1-GFP foci share other properties of the cSGs. Firstly, as cSGs are characterized by rapid disassembly when the stress ceases, the fate of the SCO1-GFP foci was examined during heat recovery. After subjecting the seedlings to heat stress, the plants were moved back to the control conditions, and SCO1-GFP localization was followed in a time-course manner. SCO1-GFP1 foci formed within 30 min of heat stress and disassembled 40 min into stress recovery (Figure 1A). Secondly, the formation of cSGs is abolished by cycloheximide, a small-molecule inhibitor of protein translation. Analogously, we decided to evaluate whether ccpSG formation might be abolished by the application of lincomycin (Linc), a translation inhibitor specific for plastidial machinery, known to disturb the formation of the first peptide bond from newly synthesized polypeptides (Herrin and Nickelsen, 2004; Chotewutmontri and Barkan, 2018). Treatment with Linc alone did not induce cpSG formation, suggesting that disruption of the translation machinery at the early stages of translation does not cause mRNA aggregation. However, incubation with Linc *prior* to the heat stress prevented the assembly of the SCO1-GFP foci (Figure 1B). This made us conclude that, even though the mechanism of action for Linc is different to cycloheximide, disruption of peptide extension at the initiation stage is enough to prevent mRNA sequestration into cpSG under heat stress conditions. Next, to determine whether, similarly to cSGs, SCO1-GFP foci are composed of a fluid shell surrounding a stable core (Wheeler et al., 2016), we used a previously described treatment with 10% 1,6-hexanediol (Peskett et al., 2018), which interferes with the weak hydrophobic protein–protein and protein–RNA interactions characteristic for the SGs shell (Patel et al., 2007; Kroschwald et al., 2015; Molliex et al., 2015). Directly after the addition of 1,6-hexanediol to the lysate prepared from the heat-treated SCO1-GFP seedlings, the SCO1-GFP foci were examined with a confocal microscope by a collection of time frames for 5 min, as described in Peskett et al. (2018). The 1,6-hexanediol treatment decreased the size of SCO1-GFP foci by approximately 30% (Figures 2A,B and Supplementary Table S2). These results demonstrated that SCO1-GFP foci are composed of a 1,6-hexanediol-sensitive shell and a 1,6-hexanediol-resistant and stable core. Finally, to determine what threshold temperature is required for the assembly of SCO1-GFP foci, SCO1-GFP seedlings were exposed to different temperature and time treatments. While we observed no SCO1-GFP foci formation at 27 and 30°C, 30 min of 33°C treatment was sufficient to induce the appearance of SCO-1-GFP foci (Figure 2C and Supplementary Figure S2). Similarly, cSGs were shown to form in temperatures above 34°C (Hamada et al., 2018). In summary, these results support the notion of SCO1-GFP foci having SG-like properties. From this point, we will refer to the SCO1-GFP foci as plastidial SGs (cpSG).

### Protein and Metabolite Composition of the cpSG

**FIGURE 3:**
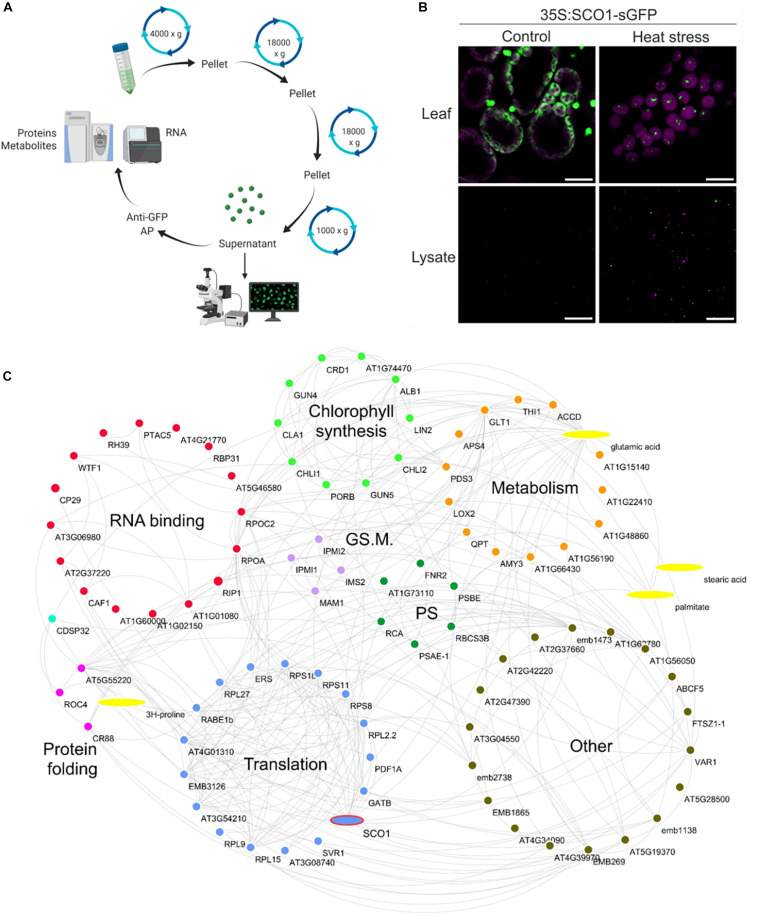
Stress granule isolation. **(A)** Graphical representation of ccpSG isolation protocol. The figure was prepared using Biorender.com. **(B)** Cellular localization of SCO1-GFP in *Arabidopsis* seedlings subjected to control and heat/dark stress (30 min at 42°C, darkness) treatment. The upper panel corresponds to intact leaves, while the lower panel corresponds to SG-enriched lysate used as input for affinity purification steps with anti-GFP antibody. Scale bars = 10 μm. **(C)** Proteome and metabolome composition of cpSG. Visualization of cpSG proteome and metabolome by using Cytoscape (Shannon et al., 2003). Proteins and metabolites are represented as nodes. Edges were imported from the STITCH database (Szklarczyk et al., 2016) using experimental, database, and literature evidence. The color code for the nodes corresponds to the biological process that the proteins are involved in. Biological processes were manually assigned based on the information from UniProt (Apweiler et al., 2004). PS, photosynthesis; GS. M., glucosinolate metabolism.

In order to demarcate the protein and metabolite composition of the cpSG, we used SCO1-GFP seedlings grown in a liquid medium and subjected to either control conditions or a combination of heat and dark treatment (30 min, 42°C, and darkness). Following a previously published cSG isolation protocol (Kosmacz et al., 2019; Figure 3A), cpSG were enriched using a series of differential centrifugations, yielding a SG-enriched lysate that was used as input for affinity purification (AP) with anti-GFP antibodies and dynabeads. As observed before, under control conditions, SCO1-GFP was localized uniformly in the plastidial stroma, whereas under stress conditions, SCO1-GFP was sequestered into cpSG (Figure 3B). Importantly, no SCO1-GFP foci could be observed in the SG-enriched lysate from the control plants (Figure 3B). Purified cpSG, also referred to as cpSG isolates, were subjected to mass spectrometry (MS)-based proteomics and metabolomics analysis. A separate isolation was performed to search for cpSG-associated RNAs by extraction of all RNAs from purified cpSG followed by Illumina RNA-sequencing. As an additional negative control, we used seedlings over-expressing plastidial glyceraldehyde-3-phosphate dehydrogenase (35S:GAPCP1-GFP) (Munoz-Bertomeu et al., 2009). Similarly to SCO1-GFP, GAPCP1-GFP assembles into dot-like foci (Supplementary Figure S3), but unlike the SCO1-GFP, GAPCP1-GFP foci are already present under optimal conditions. GAPCP1-GFP foci were isolated using the SG isolation protocol, with 35S:GFP lines used as a control to exclude non-specific interactors.

Multiple criteria were used to define cpSG-associated proteins and metabolites (Supplementary Tables S3–S5). As the cpSG were only present in the heat/dark-treated seedlings, we first selected for proteins and metabolites that were either (i) present in the cpSG isolate of stress-treated seedlings and absent in the cpSG isolate of control seedlings or (ii) significantly enriched (fold change > 2; *t*-test, *p*-value < 0.05) in the cpSG isolate from the stress-treated *versus* the control seedlings. We then excluded a total of 31 cytosolic, nuclear, mitochondrial, and membrane proteins, as these are unlikely to localize into plastidial SGs and can therefore be viewed as false positives. Subcellular localization was retrieved from the SUBA database (Hooper et al., 2017). Notably, the exclusion list contained 13 of the 118 proteins previously identified in the cSGs, e.g., polyadenylate-binding protein 8, eukaryotic translation initiation factor 5A-2, and ribonuclease TUDOR-1 (Kosmacz et al., 2019). Such overlap corroborates the previously raised possibility of cpSG and cSGs fusing during lysate preparation. The obtained list comprised 88 proteins and four metabolites (Figure 3C). Notably, of the 88 cpSG proteins, 14 were among the 28 plastidial proteins identified in the cSG isolates (Kosmacz et al., 2019). Considering that the measured plastidial proteome contains approximately 1,500 proteins (Zybailov et al., 2008), such overlap is 8.5 times more than expected by chance and highly significant (Fisher exact test, *p*-value = 0.0006). Finally, the list of 88 cpSG proteins was compared with the list of 86 plastid proteins (Supplementary Table S4) associated with the 35S:GAPCP1-GFP condensate (negative list). The comparison revealed an overlap of eight proteins. Considering that the measured plastidial proteome contains approximately 1,500 proteins (Zybailov et al., 2008), such overlap is again 1.6 times more than expected by chance, but the enrichment is not significant (Fisher exact test, *p*-value = 1), attesting to the specificity of the cpSG proteome presented here.

The sequence analysis (Lancaster et al., 2014) of the 88 ccpSG proteins revealed the presence of two proteins with prion-like intrinsically disorganized regions associated with SG assembly: CP29A is an RNA-binding protein, and RIP1 (MORF 8) is an RNA editing factor found in plastids and mitochondria. Five of the 88 ccpSG proteins including CP29A have RNA recognition motifs similar to those present in proteins involved in the nucleation of the cSG protein–RNA scaffolds. In fact, CP29 is a paralog of the cSG marker protein RBP47b. Additionally, 15 of the 88 proteins have nucleoside triphosphate hydrolase activity, three are chaperones, 11 constitute ribosomal subunits, and SCO1 and RABE1b are translational elongation factors (Figure 3C).

In addition to proteins which, based on what is known for cSGs, can be linked with cpSG assembly (e.g., CP29A), dynamics (e.g., ATPases), and function (e.g., chaperones) (Kosmacz et al., 2019), the cpSG interactome was characterized by the presence of numerous metabolic enzymes. Chlorophyll biosynthesis clearly stood out (Figure 3C). In addition to all three subunits of the magnesium chelatase complex (ALB1, GUN5, and CHLI1), cpSG sequestered additional enzymes and regulators involved in chlorophyll accumulation, including the magnesium chelatase-associated proteins GUN4 and CHLI2 and an enzyme downstream of the magnesium chelatase complex, PORB. The list of cpSG proteins also contained Rubisco activase and Rubisco accumulation factors required for Rubisco activity. Intriguingly, the enzymes responsible for the different steps of glucosinolate synthesis were found in both cSGs (Kosmacz et al., 2019) and cpSG (here).

**FIGURE 4:**
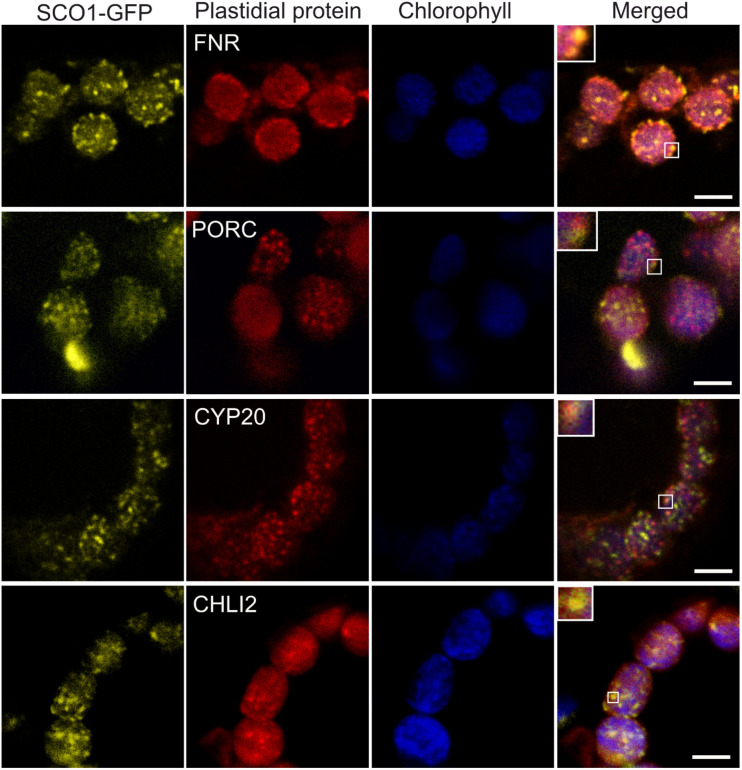
Co-localization of SCO1-GFP with the RFP-tagged SCO1 interactors under heat stress conditions. The yellow channel corresponds to signals from stably transformed SCO1-GFP *Arabidopsis* plants. Red corresponds to transiently expressed, RFP-tagged plastidial proteins FNR, PORC, CYP20, and CHLI2. The blue channel represents the autofluorescence of chlorophyll. The last channel represents a merge between all and zoomed squares on a single cpSG. Three leaves from five independently growing plants were *Agrobacterium*-infiltrated with each of the constructs. Scale bar = 5 μm.

To confirm our proteomics results, we chose four proteins identified in cpSG: FNR2 (At1g20020), PORC (At1g03630), CYP20-3 (At3g62030), and CHLI2 (At5g45930). The C-terminal/RFP fusions of the four proteins expressed under control of the constitutive CaMV 35S promoter were transiently infiltrated into the leaves of the stable *Arabidopsis* 35S:SCO1-GFP marker line. To assess whether selected proteins localize into cpSG, infiltrated leaves were treated with 42°C heat stress for 30 min *prior* to the analysis with confocal microscopy. All four proteins were present in the punctate foci, reminiscent of the cpSG (Figure 4). It is important to note that not all the recorded foci co-localized with the SCO1-GFP. Based on what is known for cSGs (Buchan and Parker, 2009), this is likely reflective of the compositional heterogeneity present in the cpSG population.

In addition to proteins, the list of cpSG constituents included four metabolites: two fatty acids, stearic acid and palmitic acid, and two amino acids, glutamic acid and proline. Both proline and glutamic acid were also identified in cSGs (Kosmacz et al., 2019). Moreover, the cpSG localization of all four metabolites can be traced to the cpSG localization of their corresponding protein interactors, e.g., proline was shown to bind to chaperones (Diamant et al., 2001).

Finally, the RNA-seq analysis of cpSG revealed the presence of plastidial transcripts (Supplementary Table S6) with highest enrichment measured for ribosomal proteins and subunits of the ATP synthase complex.

## Discussion

Stress granules are evolutionarily conserved aggregates of proteins, and untranslated mRNAs formed in response to stress. Here we demonstrate that SG-like foci also assemble in the chloroplasts of higher plants. Our supposition is based on multiple lines of evidence proving the broad similarity of the cpSG reported here with previously identified cSGs (Protter and Parker, 2016; Chantarachot and Bailey-Serres, 2018; Youn et al., 2019): (i) cSG formation is a rapid, stress-dependent, and reversible process. Analogously, we demonstrated that cpSG form within 10 min of 42°C heat stress but are absent under control conditions. Moreover, cpSG disassemble in plants transferred to control temperature, 40 min into the heat stress recovery. We also showed that, similarly to cSGs, cpSG are induced by temperatures higher than 33°C (Hamada et al., 2018); (ii) Cycloheximide treatment is known to interfere with the formation of cSGs by trapping mRNAs required for the assembly of cpSG/cSGs scaffolds at polysomes (Kedersha et al., 1999; Sorenson and Bailey-Serres, 2014). Although Lync has a different mode of action in comparison to cycloheximide, its application prevented cpSG formation under heat stress conditions; (iii) Evaluation of cpSG properties by hexanediol treatment (Wheeler et al., 2016; Peskett et al., 2018) revealed that, similarly to cSGs, cpSG belong to the so-called liquid–solid demixing phase separation assemblies, which are composed of a stable core and a fluid shell. While a core is composed of a dense network of protein–protein-mRNA interactions, the shell is less concentrated and more dynamic in terms of its protein composition (Jain et al., 2016; Youn et al., 2018; Kosmacz et al., 2019).

As demarcated here, the cpSG proteome and metabolome bear a functional resemblance with those reported for cSGs. Remarkably, this resemblance extends not only to plant cSGs (Jain et al., 2016; Youn et al., 2018; Kosmacz et al., 2019) but also to SGs in yeast and mammalian cells (Jain et al., 2016; Youn et al., 2018; Kosmacz et al., 2019), attesting to the evolutionary conservation of proteins required for SG formation, dynamics, and function. RNA-binding proteins with prion-like domains, homologous to the CP29A reported here, are essential scaffold proteins because of their ability to form polymers and to sequester mRNAs (Lorkovic and Barta, 2002). Indeed CP29A was shown to bind and regulate the stability of multiple chloroplast mRNAs (Kupsch et al., 2012). This makes CP29A a key candidate protein for cpSG assembly and function. Moreover, an important role in the regulation of cSG dynamics has been assigned to proteins with ATPase activity (Jain et al., 2016), as ATP hydrolysis is required for stress granule assembly and dynamics. Enzymes with ATPase activity, e.g., DEAD box RNA helicase (RH3), were also found in cpSG. Interestingly, we also identified protoporphyrinogen IX oxidase, an enzyme from the tetrapyrrole biosynthetic pathway which was also reported to play an additional role in RNA editing of plastidial transcripts, leading to the modulation of NADH dehydrogenase-like complex activity (Zhang et al., 2014). A detailed analysis of RNA present in cpSG revealed the presence of two, ATCG00905 and ATCG01230, transcripts identified as edited targets by MORF2 (through induction by GUN1) (Zhao et al., 2019), which suggests that cpSG might be also formed as foci supporting RNA editing events in chloroplasts in the context of retrograde signaling. Finally, chaperones identified in cpSG, e.g., heat shock protein 90-5, likely function to prevent the misfolding of proteins concentrated in SGs. Along similar lines, proline that accumulates in cSGs and cpSG was shown to activate molecular chaperones and, by doing so, may prevent protein misfolding (Diamant et al., 2001).

Our proteomics analysis also revealed the presence of multiple stress-related proteins in *Arabidopsis* cpSG. The prime function of cSGs is to protect proteins from unfolding and aggregation. We speculate that cpSG may have a similar role. To exemplify this, we will use translation elongation factor Tu (RABE1b), which we detected in purified cpSG. RABE1b was shown to be prone to heat-induced irreversible aggregation both *in vitro* and *in vivo*, but while *in vitro* RABE1b aggregation could be observed even under slightly elevated temperatures, *in vivo* it required much higher temperatures, above 40°C (Li et al., 2018). The authors speculated about the existence of a cellular mechanism that would promote RABE1b stability and reduce its aggregation. Such a mechanism would be crucial because RABE1b is essential for plastid translation and heat stress tolerance. We speculate that reversible sequestration of RABE1b into cpSG, as reported here, would confer previously demonstrated *in vivo* resistance to unfolding and aggregation. Similarly, this could be true for Rubisco activase, also found in cpSG, which is extremely sensitive to aggregation by heat stress (Salvucci and Crafts-Brandner, 2004).

In addition to its role in conferring protection, cSG sequestration was also shown to serve as a mechanism to temporarily deactivate regulatory proteins important, e.g., for plant growth (Jain et al., 2016; Youn et al., 2018; Kosmacz et al., 2019). We propose that such a mechanism could be relevant for chlorophyll biosynthetic enzymes such as the magnesium chelatase complex (Gibson et al., 1995; Papenbrock et al., 2000; Ikegami et al., 2007), which were identified in cpSG. Because of their chemical reactivity, the levels of both chlorophyll and chlorophyll intermediates need to be tightly controlled. Rapid inactivation of chlorophyll synthesis by cpSG sequestration of the key biosynthetic enzymes offers an elegant mechanism to regulate chlorophyll levels in response to changing environmental conditions, such as heat and darkness imposed in our experiment. Once the stress ceases, cpSG disassemble, and chlorophyll synthesis could be quickly reactivated to meet photosynthetic demand.

Similarly to protein, mRNA sequestration within SGs can have a double role. On one hand, it protects mRNAs from degradation and, on the other hand, it can be used as means of translational regulation. For instance, under low-oxygen stress, cSG sequestration of the mRNAs encoding abundant, non-stress transcripts contributes to the preferential translation of stress-induced mRNAs. Upon reoxygenation, cSGs disassemble and the stored mRNAs return to polysomes (Sorenson and Bailey-Serres, 2014).

Here we present a compelling evidence for the existence of cpSG in higher plants. We demonstrated that cpSG sequester a plethora of proteins, metabolites, and mRNAs. By drawing a homology with cSGs, it is fair to speculate about the biological role of cpSG sequestration, on one hand as a protection mechanism and on the other hand as a means of rapid regulation. Future work will focus on (i) understanding the exact mechanism underlying cpSG assembly and disassembly, (ii) assessing compositional differences related to varying stress conditions, and, most importantly, (iii) validation of the biological significance of cpSG sequestration.

## Data Availability Statement

The datasets presented in this study can be found in online repositories. The names of the repository/repositories and accession number(s) can be found in the article/ Supplementary Material.

## Author Contributions

AS and MC conceived the project and wrote the manuscript. MC, AN-D, AN, JM, AM, and ES performed experimental work. AN-D and AN were involved in review and editing. AS provided resources. AS and MC agreed to serve as the authors responsible for contact and ensure communication. All authors contributed to the article and approved the submitted version.

## Conflict of Interest

The authors declare that the research was conducted in the absence of any commercial or financial relationships that could be construed as a potential conflict of interest.
