# Supplementary material for: Identification and Characterization of the Heat-Induced Plastidial Stress Granules Reveal New Insight Into Arabidopsis Stress Response
Source: Front Plant Sci. 2020 Oct 30;11:595792. doi: 10.3389/fpls.2020.595792 (PMC7674640; doi:10.3389/fpls.2020.595792)
Supplement: Supplementary file 2 [file Data_Sheet_2.docx]

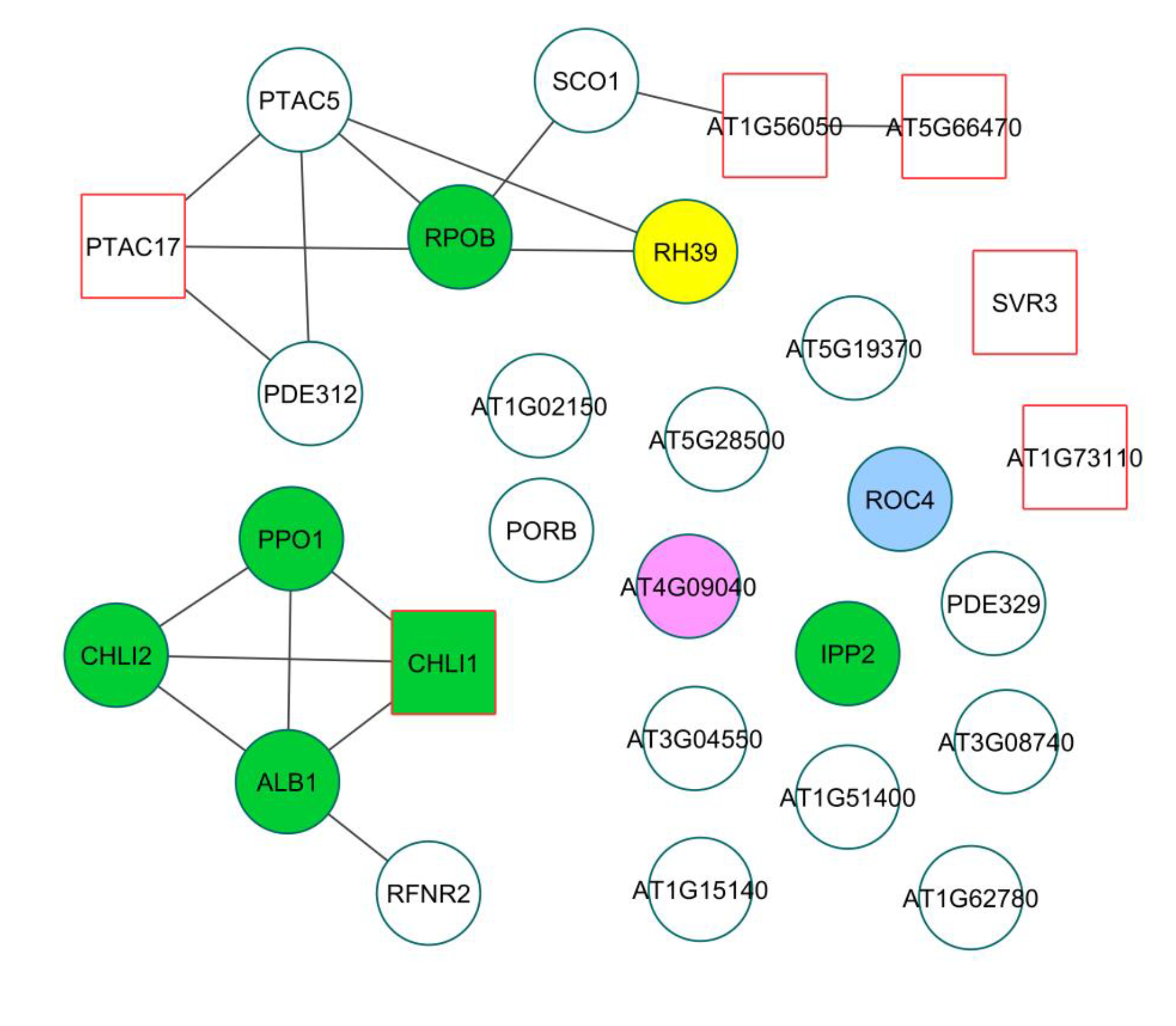


**Figure S1.** **Interaction network of the 28 plastidial proteins identified in the cSGs isolates (Kosmacz et al., 2019).** Visualization was done using Cytoscape (Shannon et al., 2003). Proteins are represented as nodes. Edges were imported from the STITCH database (Szklarczyk et al., 2016) using experimental, database, and literature evidence. With green indicated chlorophyll biosynthetic enzymes, with square shape proteins with reported nucleoside triphosphate hydrolase activity, with blue shading chaperone, with yellow RNA helicase, with pink RNA binding protein with RRM motifs characteristic for the cSGs scaffold proteins such as e.g. Rbp47b.


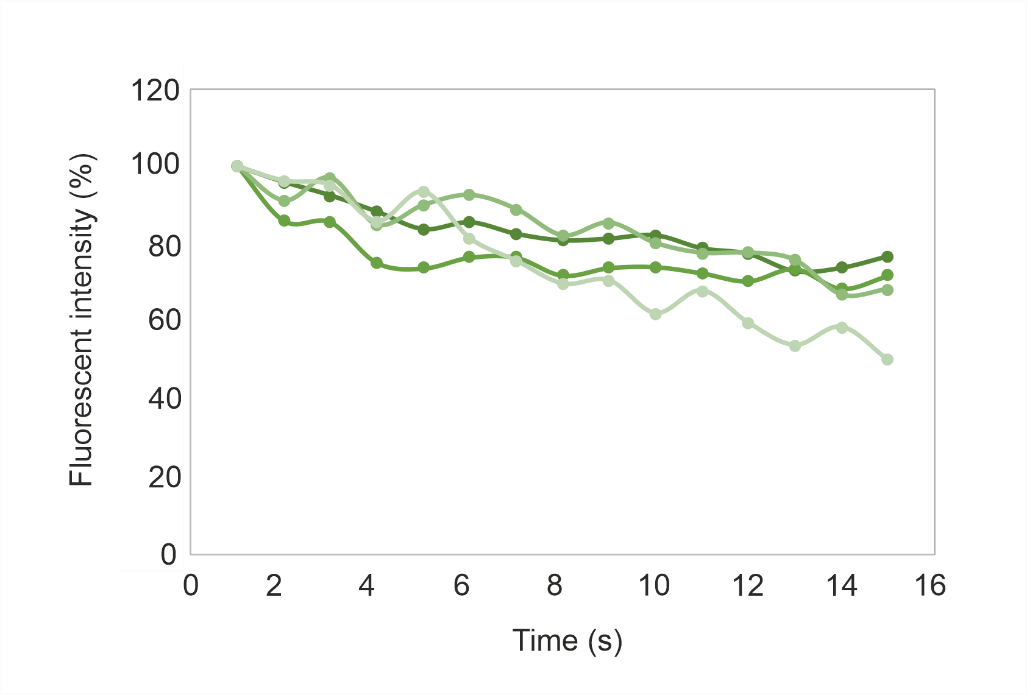


**Figure S2.** **Quantification of fluorescence intensity of SCO1-GFP foci after addition of 10% 1,6-hexanediol (replicates from experiment presented on Figure 2, measurements included in the Table S2)**. Measurements were performed on random cpSG from four different lysates.

**
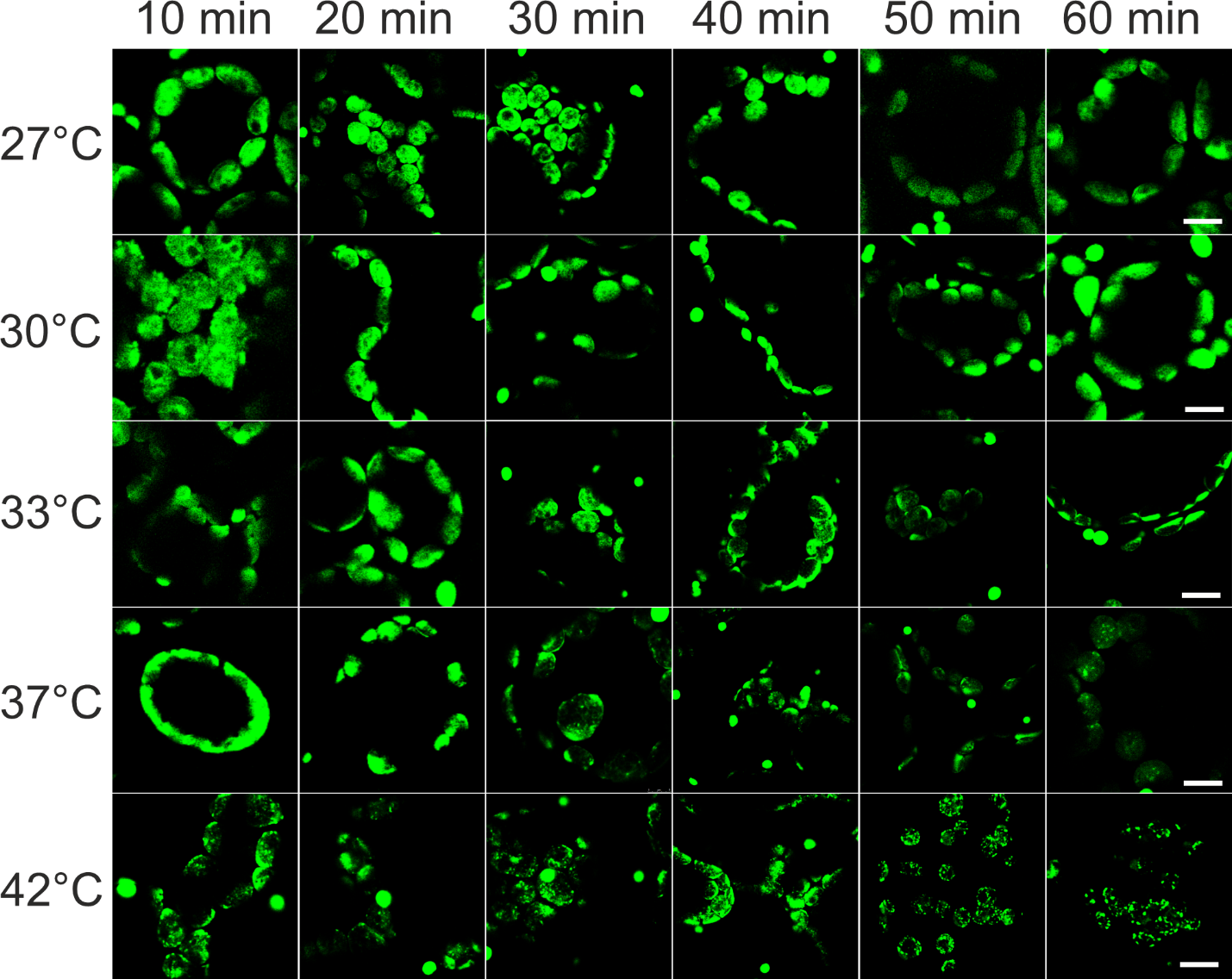
**

**Figure S3. Micrographs represent time and temperature dependent cpSG formation.** Localization was followed under confocal microscope using Arabidopsis 10-day old seedlings expressing SCO1-GFP marker. Scale bar = 10 µm.


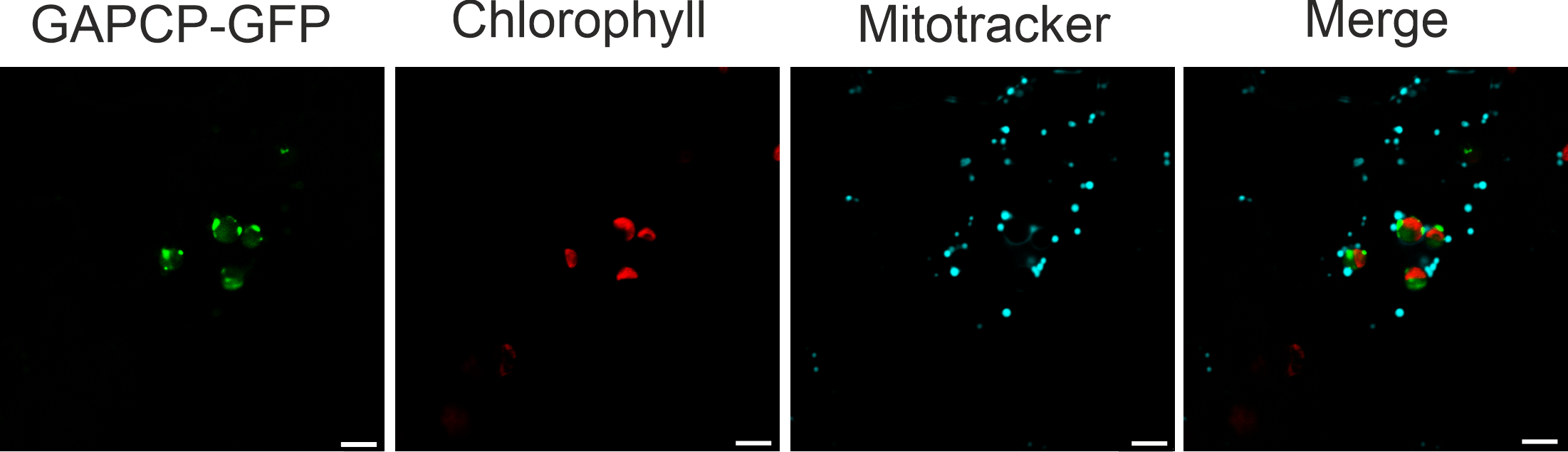


**Figure S4. GAPCP localization.** GAPCP-GFP Arabidopsis seedlings were used to follow GAPCP localization. Scale bar = 20 µm.

**Supplementary material**

**Table S1. List of plastidial proteins identified in the cSGs isolates.** Data were retrieved from Kosmacz et al., 2019. Given are: TAIR identifier (A), FASTA header (B), number of unique peptides used for protein identification (C), Log2 normalized LFQ (label free quantification) intensities used for data analysis (D-H), fold changeof protein abundance (J) calculated between stress and control conditions was used to deliniate cSGs proteins. Data are from three cSGs isolations.

**Table S2. Intensity measurment per area of cpSG after 1,6-hexanediol treatment.** First replicate is included on Figure 2a-b, four replicates are included on **Figure S2**.

**Table S3. List of proteins identified in the cpSGs isolates.** Given are: Protein identifier (A, C), FASTA header (B), consensus subcellular localisation retrieved from SUBA (D), number of unique peptides used for protein identification (E), raw intensities used for qualitative data analysis (F-L), Log2 normalized LFQ (label free quantification) intensities used for quantitative data analysis (N-T), cpSGs proteins identified based on presence ver. absence criteria using raw intensities (M), fold change (V) and unpaired, two tailed TTEST (U) calculated between stress and control conditions was used to deliniate cpSGs proteins (using Log2 normalized LFQ intensities). Data are from 3-4 cpSGs isolations. Also indicated proteins identified in the GAPCP pull-down used as a negative control (X).

**Table S4. List of proteins identified in the GAPCP1 isolates.** Given are: Protein identifier (A, C), FASTA header (B), consensus subcellular localisation retrieved from SUBA (D), number of unique peptides used for protein identification (E), raw intensities used for qualitative data analysis (F-M), Log2 normalized LFQ (label free quantification) intensities used for quantitative data analysis (O-V), GAPCP1 foci proteins identified based on presence ver. absence criteria using raw intensities (N), fold change (X) and unpaired, two tailed TTEST (W) calculated between GAPCP1:GFP and 35S:GFP isolates was used to deliniate GAPCP1 foci proteins (using Log2 normalized LFQ intensities). Data are from 4 isolations.

**Table S5. List of metabolites identified in the cpSGs isolates.** Log2 normalized intensities used for data analysis (E-L). Fold change and unpaired, two tailed TTEST calculated between stress and control conditions was used to deliniate cpSGs metabolites.

**Table S6. Transcripts identified by RNA-sequencing cpSG isolates.**

**Hooper CM, Castleden IR, Tanz SK, Aryamanesh N, Millar AH** (2017) SUBA4: the interactive data analysis centre for Arabidopsis subcellular protein locations. Nucleic Acids Res **45:** D1064-D1074

**Kosmacz M, Gorka M, Schmidt S, Luzarowski M, Moreno JC, Szlachetko J, Leniak E, Sokolowska EM, Sofroni K, Schnittger A, Skirycz A** (2019) Protein and metabolite composition of Arabidopsis stress granules. New Phytol **222:** 1420-1433

**Shannon P, Markiel A, Ozier O, Baliga NS, Wang JT, Ramage D, Amin N, Schwikowski B, Ideker T** (2003) Cytoscape: a software environment for integrated models of biomolecular interaction networks. Genome Res **13:** 2498-2504

**Szklarczyk D, Santos A, von Mering C, Jensen LJ, Bork P, Kuhn M** (2016) STITCH 5: augmenting protein-chemical interaction networks with tissue and affinity data. Nucleic Acids Res **44:** D380-D384
